# Potentially modifiable predictors of adverse neonatal and maternal outcomes in pregnancies with gestational diabetes mellitus: can they help for future risk stratification and risk-adapted patient care?

**DOI:** 10.1186/s12884-019-2610-2

**Published:** 2019-12-04

**Authors:** Maria-Christina Antoniou, Leah Gilbert, Justine Gross, Jean-Benoît Rossel, Céline J. Fischer Fumeaux, Yvan Vial, Jardena J. Puder

**Affiliations:** 1 Pediatric Service, Department Woman-Mother-Child, Lausanne University Hospital, Lausanne, Switzerland; 2 Obstetric Service, Department Woman-Mother-Child, Lausanne University Hospital, Lausanne, Switzerland; 3 Service of Endocrinology, Diabetes and Metabolism, Lausanne University Hospital, Lausanne, Switzerland

**Keywords:** Gestational diabetes, HbA1c, Pregnancy outcomes, Risk stratification

## Abstract

**Background:**

Gestational diabetes mellitus (GDM) exposes mothers and their offspring to short and long-term complications. The objective of this study was to identify the importance of potentially modifiable predictors of adverse outcomes in pregnancies with GDM. We also aimed to assess the relationship between maternal predictors and pregnancy outcomes depending on HbA1c values and to provide a risk stratification for adverse pregnancy outcomes according to the prepregnancy BMI (Body mass index) and HbA1c at the 1st booking.

**Methods:**

This prospective study included 576 patients with GDM. Predictors were prepregnancy BMI, gestational weight gain (GWG), excessive weight gain, fasting, 1 and 2-h glucose values after the 75 g oral glucose challenge test (oGTT), HbA1c at the 1st GDM booking and at the end of pregnancy and maternal treatment requirement. Maternal and neonatal outcomes such as cesarean section, macrosomia, large and small for gestational age (LGA, SGA), neonatal hypoglycemia, prematurity, hospitalization in the neonatal unit and Apgar score at 5 min < 7 were evaluated. Univariate and multivariate regression analyses and probability analyses were performed.

**Results:**

One-hour glucose after oGTT and prepregnancy BMI were correlated with cesarean section. GWG and HbA1c at the end pregnancy were associated with macrosomia and LGA, while prepregnancy BMI was inversely associated with SGA. The requirement for maternal treatment was correlated with neonatal hypoglycemia, and HbA1c at the end of pregnancy with prematurity (all *p* < 0.05). The correlations between predictors and pregnancy complications were exclusively observed when HbA1c was ≥5.5% (37 mmol/mol). In women with prepregnancy BMI ≥ 25 kg/m^2^ and HbA1c ≥ 5.5% (37 mmol/mol) at the 1st booking, the risk for cesarean section and LGA was nearly doubled compared to women with BMI with < 25 kg/m^2^ and HbA1c <  5.5% (37 mmol/mol).

**Conclusions:**

Prepregnancy BMI, GWG, maternal treatment requirement and HbA1c at the end of pregnancy can predict adverse pregnancy outcomes in women with GDM, particularly when HbA1c is ≥5.5% (37 mmol/mol). Stratification based on prepregnancy BMI and HbA1c at the 1st booking may allow for future risk-adapted care in these patients.

**Electronic supplementary material:**

The online version of this article (10.1186/s12884-019-2610-2) contains supplementary material, which is available to authorized users.

## Background

Gestational Diabetes Mellitus (GDM) is defined as diabetes first diagnosed in the second or third trimester of pregnancy that is not preexisting, type 1 or type 2 diabetes [1]. The incidence of GDM has dramatically increased during the past decades reflecting the ongoing epidemic in obesity and type 2 diabetes [2, 3]. In Switzerland, using the International Association of Diabetes and Pregnancy Study Group (IADPSG) Criteria, GDM is diagnosed in 10.9% of pregnancies [4]. GDM exposes the mothers and their offspring to short and long-term complications [4–7]. Previous studies have shown the beneficial effect of GDM treatment on neonatal outcomes such as birth weight and fat, large for gestational age (LGA) and shoulder dystocia, as well as maternal outcomes, including cesarean section and preeclampsia [8, 9].

Several potentially modifiable predictors regarding adverse outcomes have been studied. Maternal overweight and obesity increase the risk for neonatal complications [10, 11]. In pregnancies with GDM, gestational weight gain (GWG) is associated with cesarean section and LGA [12]. The implementation of the Institute of Medicine guidelines of BMI-based weight gain [13] has also increased knowledge about the deleterious effect of excess gestational weight gain (EWG) on neonatal outcomes [14–16] . Nevertheless, as far as we know, there is no study evaluating whether GWG or EGW is a more potent predictor of adverse neonatal outcomes.

Maternal metabolic control affects neonatal birth outcomes. The correlation between glycated hemoglobin (HbA1c) at GDM diagnosis and neonatal complications remains controversial [17–20]. Recent studies have demonstrated the association between third trimester HbA1c and adverse neonatal outcomes in offspring of treated women [21, 22]. Few studies have aimed to identify third trimester HbA1c cutoffs above which neonatal complications are increased; nevertheless, specific HbA1c cutoffs are still lacking [22–24]. Maternal glucose values during the oral glucose tolerance test (oGTT) at 24–28 weeks of gestational age have been linked to adverse neonatal outcomes in previous studies [25, 26], although their value is different in the context of a treated GDM population.

Finally, a recent study in a population with GDM, demonstrated that there is an association between the need for medical glucose-lowering treatment and adverse pregnancy outcomes including cesarean section, LGA, SGA, and prematurity [27]. On the other side, a recent Atlantic DIP study did not find any difference in neonatal hypoglycemia rates in women with GDM treated with insulin vs nutritional care [28]. Further studies are needed to evaluate whether the risk of neonatal hypoglycemia is higher if a maternal medical treatment is required in order to create evidence-based guidelines for neonatal glucose monitoring or other adverse outcomes.

The objective of this study was: 1) to identify the respective importance of potentially modifiable predictors of adverse neonatal and maternal outcomes in the context of a treated population of women with GDM. 2) to assess the relationship between these predictors and outcomes depending on HbA1c values. 3) to evaluate the impact of absolute GWG versus of EGW on these outcomes. 4) to provide a risk stratification for adverse pregnancy outcomes depending on prepregnancy Body Mass Index (BMI) and HbA1c at the 1st booking at the GDM clinic.

## Methods

### Study design and patient population

This is a prospective observational study, which included pregnant women with GDM followed in the Diabetes and Pregnancy Unit in the Centre Hospitalier Universitaire Vaudois (CHUV), Lausanne, Switzerland, between April 2012 and October 2017.

### GDM diagnosis, treatment and follow-up

GDM was diagnosed according to the IADPSG Criteria [29]. Thus, the diagnosis of GDM was confirmed if fasting blood glucose (FBG) was ≥5.1 mmol/l and/or 1 h blood glucose was ≥10.0 mmol/l and/or 2 h blood glucose was ≥8.5 mmol/l, following a 75 g oGTT. The treatment was based on the current guidelines of the American Diabetes Association [30] and of the Endocrine Society [31]. At their first clinical appointment, patients were seen by a nurse specialized in GDM or a medical doctor, received information on GDM and were taught how to perform the capillary blood glucose test. A dietician saw women 1 week later and provided them with recommendations regarding their GDM, lifestyle management and weight gain during pregnancy. Women were encouraged to increase physical activity and had the possibility to receive physical activity counseling by a physiotherapist, as well as to participate in GDM physical activity groups. According to international and local guidelines (Vaud Cantonal Diabetes Program [32, 33]), women were asked to control their capillary glucose values 4 times per day (fasting blood glucose (FBG) in the morning and 2-h (or 1-h) postprandial blood glucose after each meal). Additional glucose controls were recommended on an individual basis, if needed. If despite lifestyle changes, glucose values remained above targets two or more times during a 1 to 2-week period (FBG > 5.3 mmol/l, 1-h postprandial glucose > 8 mmol/l and 2-h postprandial glucose > 7 mmol/l), metformin or insulin treatment was introduced, depending on glucose values (i.e. insulin in case of relatively high values), patient characteristics (i.e BMI) and patient preference. Short acting insulin analogues were introduced and adapted to achieve 1-h postprandial glucose ≤8 mmol/l or 2-h postprandial glucose ≤7 mmol/l, and long acting insulin analogues to achieve FBG ≤5.3 mmol/l.

### Maternal and neonatal anthropometric and metabolic parameters

Prepregnancy Body Mass index (BMI) was calculated based on self-reported pre-pregnancy weight or retrieved from medical charts and measured height on the first visit at the GDM clinic, using the formula weight (kg)/[height(m)]^2^. First booking BMI was calculated based on the measured weight and height on the first visit at the GDM clinic. Gestational weight gain was determined as the difference between the last weight measured before delivery and pre-pregnancy weight. Excessive gestational weight gain (EGW) was defined as gestational weight gain exceeding the thresholds established by the Institute of Medicine (IOM) Guidelines 2009 for the respective maternal pre-pregnancy BMI category [13]. HbA1c levels were measured both on the 1st booking at the GDM clinic and at the end of pregnancy (last visit before delivery). HbA1c at the end of pregnancy was only performed after March 2015. Hba1c was measured using a chemical photometric method (conjugation with boronate; Afinion®). The Afinion® analyzer has shown to have similar accuracy and precision compared to the high-performance liquid chromatography (HPLC), which is IFCC (International Federation of Clinical Chemistry and Laboratory Medicine) standardized and DCCT (Diabetes Control and Complications Trial) aligned [34]. The maternal treatment was documented and classified in 2 categories (no treatment, treatment with metformin and/or insulin). Maternal ethnicity was also recorded and classified in Low (Europe, North America) and High Risk (Asia, Central and South America, Africa, Oceania) ethnic groups [30]. Cesarean section was the only maternal complication assessed by this study; the decision for cesarean section indication was taken by the mother’s obstetrician.

Neonatal growth parameters such as weight (g) and length (cm), and head circumference were documented at birth as an absolute value; percentiles and z-scores for each of the above mentioned parameters were calculated using the Intergrowth 21st newborn size application tool [35]. Large-for-gestational-age was defined as newborn weight > 90th percentile for sex and gestational age. Small-for-gestational-age birth weight was defined as newborn weight < 10th percentile for sex and gestational age. Macrosomia was defined as birth weight ≥ 4000 g. Prematurity was defined as gestational age < 37 weeks. Gestational age was calculated according to the date of the last menstruations, or as assessed by the fetal ultrasound in the cases where gestational age was corrected during the early in-utero ultrasound evaluation. According to the center protocol, all GDM neonates received feeding in the first 2 h of life and were fed every 2–3 h during the first 48 h in order to prevent neonatal hypoglycemia. Systemic blood glucose monitoring was conducted according [36] in all newborns the frequency of the controls (minimum 3) depending on whether the mother was treated or not with insulin during her pregnancy (minimum 8 controls, during 48 h in case of maternal treatment). Neonatal glycemia was also measured if symptoms suggested hypoglycemia. Hypoglycemia was defined as capillary or venous glucose value ≤2.5 mmol/l. The blood glucose value (capillary or venous) was also verified in the CHUV central laboratory if capillary glycemia measured by the glucometer was ≤2.5 mmol/l. Neonates were hospitalized for intravenous glucose infusion when they presented a symptomatic hypoglycemia, or a glycemia ≤2.0 mmol/L, or more than one hypoglycemia ≤2.5 mmol/L despite administration of additional milk.

Other assessed neonatal complications were stillbirth or death, Apgar score at 5 min < 7, jaundice requiring phototherapy and hospitalization in the neonatal unit.

Neonatal data were obtained from the center patient electronic medical chart for all newborns born in the CHUV. In the cases where delivery took place in another hospital or clinic, anthropometric parameters at birth were provided, if possible, by the mother during her 6–8 weeks post partum visit at the Diabetes and Pregnancy Unit.

### Ethics

Signed informed consent to use maternal and infant data was obtained from all participating women. Participation in the study did not interfere with the typical care that patients receive during pregnancy and after childbirth. The study was conducted in accordance with the guidelines of the declaration of Helsinki, and good clinical practice. The Human Research Ethics Committee of the Canton de Vaud approved the study protocol (326/15).

### Predictors and outcomes

The predictors in our study included the following potentially modifiable maternal anthropometric and metabolic parameters: prepregnancy BMI, GWG and EWG, fasting, 1-h and 2-h blood glucose values after the 75 g oGTT, HbA1c at the 1st booking after GDM diagnosis and at the end of the pregnancy, and maternal medical treatment requirement. Of note, correlation between prepregnancy BMI and BMI at the 1st booking after GDM diagnosis was very high (*r* = 0.93) and results unchanged when the latter was used for analysis. The pregnancy outcomes were all binary variables and included cesarean section, macrosomia, LGA, SGA, hypoglycemia, prematurity, hospitalization in the neonatal unit, and Apgar score at 5 min < 7.

### Statistical analysis

All data were analyzed using Stata/SE 15.0 (StataCorp LLC, TX, USA). Normality of continuous variables was assessed, and normally distributed continuous variables were described as means and standard deviations (SDs). Binary outcomes were described as percentages.

Univariate logistic regression analyses, adjusting for gestational age at birth, neonatal sex and maternal age, were initially conducted. Due to the small number of some neonatal complications, analysis was only performed for adverse outcomes present in more than 20 cases [37]. Therefore, the following outcomes were removed from further analysis: 5-min Apgar score < 7, stillbirth and death.

Maternal predictors of adverse neonatal outcomes with a *p*-value < 0.2 in the univariate analysis were included in the stepwise multiple logistic regression analysis model. This analysis was also adjusted for gestational age at birth, neonatal gender and maternal age, and was conducted in order to identify the maternal anthropometric and metabolic predictors that are most significantly associated with adverse neonatal and maternal outcomes. Due to the strong correlation between HbA1c at the 1st booking and at the end of pregnancy (*r* = 0.73), two separate multiple regression models were selected when these two predictors had a *p*-value < 0.2 in the univariate analysis for the same outcome. Women were then divided in 2 groups depending on their HbA1C value at the 1st booking (HbA1c < 5.5 (37 mmol/mol) and HbA1c ≥ 5.5 (37 mmol/mol)) [38]. The cutoff value used corresponded to the median HbA1c value both at the 1st GDM booking and the end of pregnancy. Comparisons between the groups were initially made using the unpaired t-test for continuous variables and the Fischer’s exact test for binary variables. Thereafter, the multiple regression analysis selected above was performed in both stratified groups. Probability analyses according to logistic regression models were used to evaluate the risk of the 2 most frequent outcomes, i.e. cesarean section and LGA based on 2 correlates that are routinely and easily available at the 1st GDM booking: prepregnancy BMI and HbA1c at 1st GDM booking. For all analyses, adjusted odds ratios (aORs) are reported along with their 95% confidence intervals (CI), and statistical significance was defined at the two-sided *α* level of 0.05, unless specified otherwise.

## Results

### Study population description

Out of a population of 851 adult women, 111 women were excluded because they did not provide an informed consent, 5 due to a known type 1 and 8 due to known type 2 diabetes. Twelve women were excluded due to a suspicion of preexistent diabetes, and 9 because they participated in an intervention clinical trial. Finally, 130 women were excluded due to multiple gestation, missing newborn sex and/or birth weight. Overall, 576 women were included in the final analysis.

Baseline maternal characteristics are shown in Table 1. Mean maternal age was 32.8 ± 5.5 years. Fifty % of the women were either overweight or obese before pregnancy. Mean gestational age at GDM diagnosis was 28.6 weeks (SD ± 3.3). Thirty-nine % women were part of a high-risk ethnic group. Mean gestational weight gain in the cohort was 12.7 kg (SD ± 6.2), with most of the weight gain taking place before the 1st booking (10.3 kg (SD ± 5.6 kg) vs 2.5 (SD ± 2.7 kg) after). Gestational weight gain exceeded the Institute of Medicine recommendations in 30% of the patients. Mean HbA1c value was 5.5% (37 mmol/mol), SD ± 0.4% (5 mmol/mol) at the 1st booking and 5.6% (38 mmol/mol), SD ± 0.4% (5 mmol/mol) at the end of pregnancy, while median values were the same at both time points (5.5% (37 mmol/mol)). A medical treatment was required in 58% of the women; 8% were treated with metformin only and 50% required insulin (3.3% in combination with metformin). Cesarean section, including planed and emergency cesarean section, was performed in 38% of the population.

**Table 1:** Descriptive maternal and neonatal characteristics

Maternal characteristics	
Age (years)	32.8 ± 5.5
Prepregnancy BMI (kg/m^2^)	26.1 ± 5.4
BMI at the 1st GDM booking (kg/m^2^)	30 ± 5.5
Gestational weight gain (kg)	12.7 ± 6.2
Gestational weight gain until the 1st GDM booking (kg)	10.3 ± 5.6
Excessive gestational weight gain^a^ n(%)	172 (30)
Fasting oGTT glucose value (mmol/l)	5.2 ± 0.8
1-h oGTT glucose value (mmol/l)	9.6 ± 2.0
2-h oGTT glucose value (mmol/l)	7.8 ± 1.9
Gestational age at the 1st GDM booking (weeks)	28.6 ± 3.3
HbA1c at the 1st GDM booking (%)	5.5 ± 0.4
(mmol/mol)	37 ± 5
Gestational age at the end of pregnancy^b^ (weeks)	35.8 ± 2.9
HbA1c at the end of pregnancy^b^ (%)	5.6 ± 0.4
(mmol/mol)	38 ± 5.0
High risk ethnicity n(%)	219 (39)
Cesarean section n(%)	212 (38)
Maternal medical treatment requirement n(%)	297 (58)
Neonatal characteristics	
Gestational age (weeks)	38.9 ± 1.9
Male n(%)	297 (52)
LGA^c^ n(%)	95 (16.5)
SGA^d^ n(%)	54 (9.4)
Macrosomia^e^ n(%)	45 (7.8)
Hypoglycemia^f^ n(%)	56 (10.7)
Prematurity^g^ n(%)	47 (8.2)
Hospitalization for neonatal complication n(%)	61 (11.6)
Jaundice requiring phototherapy n(%)	21 (3.7)
Apgar 5-min < 7 n(%)	10 (1.7)

*Abbreviations*: *BMI* Body mass index, *GDM* Gestational diabetes mellitus, o*GTT* Oral glucose tolerance test, *HbA1c* Glycated hemoglobin, *LGA* Large for gestational age, *SGA* Small for gestational age

^a^ according to the Institute of Medicine guidelines [13]

^b^ this corresponds to the last visit at the GDM clinic

^c^ LGA: birth weight > 90th percentile for sex and gestational age using the Intergrowth 21st newborn size application tool [30]

^d^ SGA: birth weight < 10th percentile for sex and gestational age using the Intergrowth 21st newborn size application tool [30]

^e^ birth weight ≥ 4000 g

^f^ capillary or venous glucose value ≤2.5 mmol/l

^g^ gestational age < 37 weeks

Regarding the neonatal characteristics and outcomes (see Table 1), mean gestational age at birth was 38.9 weeks (SD ± 1.9). Macrosomia was present in 7.8%, LGA in 16.5% and SGA in 9.4% of newborns. Hypoglycemia occurred in 10.7% and prematurity in 8.2% of newborns. Admission in the neonatal unit was required in 11.6% of cases. Jaundice requiring phototherapy was observed in 3.7% of the neonatal population and 5-min Apgar score < 7 in 1.7%. Stillbirth was seen in 2 fetuses, of whom one was excluded from the study due to missing sex and weight data. One newborn died after birth.

### Associations between predictors and neonatal and maternal outcomes

The results of the univariate and multiple regression analyses are shown in Table 2 (univariate analyses, only those with *p* < 0.2. The integral table with all variables is shown in Additional file 1: Table S1) and Table 3 (multiple regression analyses, only those with *p* < 0.05. The integral table with all variables is shown in Additional file 2: Table S2).

**Table 2:** Maternal predictors of adverse neonatal and maternal outcomes in univariate analysis

Presence of neonatal and maternal outcomes	Maternal predictors	Odds Ratio	95% CI		*P*-value
Cesarean section	Prepregnancy BMI (kg/m^2^)	1.06	1.02	1.09	0.001
	Excess weight gain^a^	1.18	1.00	1.38	0.048
	1-h oGTT glucose (mmol/l)	1.10	0.99	1.23	0.084
	2-h oGTT glucose (mmol/l)	1.08	0.97	1.20	0.166
	HbA1c at the 1st GDM booking (%/mmol/mol)	1.63	1.04	2.56	0.033
	Maternal medical treatment requirement	1.65	1.11	2.44	0.013
Macrosomia^b^	Gestational weight gain (kg)	1.11	1.05	1.17	< 0.001
	Excess weight gain)^a^	1.41	1.10	1.79	0.006
	Fasting oGTT glucose (mmol/l)	1.63	1.16	2.29	0.005
	1-h oGTT glucose (mmol/l)	1.17	0.96	1.42	0.115
	2-h oGTT glucose (mmol/l)	1.21	0.99	1.47	0.065
	HbA1c at the 1st GDM booking (%/mmol/mol)	2.92	1.42	6.03	0.004
	HbA1c at the end of pregnancy (%/mmol/mol)^c^	6.45	1.82	22.90	0.004
	Maternal medical treatment requirement	2.75	1.29	5.86	0.009
LGA^d^	Prepregnancy BMI (kg/m^2^)	1.04	1.00	1.08	0.064
	Gestational weight gain (kg)	1.08	1.04	1.12	< 0.001
	Excess weight gain^a^	1.46	1.23	1.74	< 0.001
	Fasting oGTT glucose (mmol/l)	1.46	1.12	1.91	0.006
	1-h oGGT glucose (mmol/l)	1.13	0.99	1.29	0.077
	HbA1c at the 1st GDM booking (%/mmol/mol)	1.84	1.08	3.14	0.025
	HbA1c at the end of pregnancy (%/mmol/mol)^c^	2.84	1.01	7.93	0.047
	Maternal medical treatment requirement	1.91	1.14	3.20	0.014
SGA^e^	Prepregnancy BMI (kg/m^2^)	0.95	0.81	1.00	0.077
	Maternal medical treatment requirement	0.59	0.32	1.08	0.087
Hypoglycemia^f^	Excess weight gain^a^	1.20	0.96	1.50	0.107
	Maternal medical treatment requirement	2.03	1.06	3.88	0.032
Prematurity^g^	Gestational weight gain (kg)	0.97	0.94	1.01	0.159
	1-h oGTT glucose (mmol/l)	1.13	0.94	1.36	0.188
	HbA1c at the end of pregnancy (%/mmol/mol)^c^	12.48	1.85	84.13	0.010
Hospitalization for neonatal complication	Gestational weight gain (kg)	0.97	0.93	1.00	0.082
Jaundice requiring phototherapy	Prepregnancy BMI (kg/m^2^)	1.08	0.99	1.18	0.071
	Excess weight gain^a^	1.31	0.89	1.95	0.174

Univariate logistic regression analyses adjusted for maternal age, neonatal sex and gestational age. This table shows the odds ratio for the presence (vs absence) of adverse neonatal and maternal outcomes. Only variables with a p-value of < 0.2 are displayed. See Additional file 1: Table S1 for all results

*Abbreviations*: *CI* Confidence interval, *BMI* Body mass index, *GDM* Gestational diabetes mellitus, o*GTT* Oral glucose tolerance test, *HbA1c* Glycated hemoglobin, *LGA* Large for gestational age, *SGA* Small for gestational age

^a^ according to the Institute of Medicine 2009 guidelines [13]

^b^ birth weight ≥ 4000 g

^c^ this corresponds to the last visit at the GDM clinic

^d^ LGA: birth weight > 90th percentile for sex and gestational age using the Intergrowth 21st newborn size application tool [30]

^e^ SGA: birth weight < 10th percentile for sex and gestational age using the Intergrowth 21st newborn size application tool [30]

^f^ capillary or venous glucose value ≤2.5 mmol/l

^g^ gestational age < 37 weeks

**Table 3:** Maternal predictors of adverse neonatal and maternal outcomes in stepwise multiple logistic regression analysis

		Odds Ratio	95% CI		*P*-value
Cesarean section					
1-h oGTT-glucose (mmol/l)		1.15	1.02	1.29	0.024
Prepregnancy BMI (kg/m^2^)		1.05	1.01	1.09	0.022
Macrosomia^a^					
Gestational weight gain (kg)		1.11	1.05	1.19	0.001
HbA1c at the end of pregnancy^b^ (%/mmol/mol)		6.84	1.53	30.54	0.012
LGA^c^					
Gestational weight gain (kg)		1.11	1.06	1.17	< 0.001
HbA1c at the end of pregnancy^c^ (%/mmol/mol)		4.68	1.27	17.28	0.021
SGA^d^					
Prepregnancy BMI (kg/m^2^)		0.93	0.88	0.99	0.038
Hypoglycemia^e^					
Maternal medical treatment requirement		2.03	1.06	3.88	0.032
Prematurity^f^					
HbA1c at the end of pregnancy^c^ (%/mmol/mol)		22.4	2.36	213.2	0.007

Stepwise multiple logistic regression analyses with all the variables presented in Table 2, adjusted for maternal age, neonatal sex and gestational age. Outcomes are only shown if at least one predictor is found. Only significant results are displayed (defined significance, *P* < 0.05, see text). See Additional file 2: Table S2 for all results

*Abbreviations*: *CI* Confidence interval, *BMI* Body mass index, *GDM* Gestational diabetes mellitus, o*GTT* Oral glucose tolerance test, *HbA1c* Glycated hemoglobin, *LGA* Large for gestational age, *SGA* Small for gestational age

^a^ birth weight ≥ 4000 g

^b^ this corresponds to the last visit at the GDM clinic

^c^ LGA: birth weight > 90th percentile for sex and gestational age using the Intergrowth 21st newborn size application tool [6]

^d^ SGA: birth weight < 10th percentile for sex and gestational age using the Intergrowth 21st newborn size application tool [6]

^e^ capillary or venous glucose value ≤2.5 mmol/l

^f^ gestational age < 37 weeks

We found the following results in the multiple regression analyses: prepregnancy BMI was positively associated with cesarean section requirement *(p =* 0.022) and inversely with SGA *(p =* 0.038). Higher GWG was associated with macrosomia and LGA (all *p ≤* 0.001). The 1-h oGTT glucose value predicted cesarean section requirement (*p =* 0.024), but otherwise there was no significant association between oGTT values and adverse outcomes. Importantly, HbA1c at the 1st booking was positively associated with macrosomia, LGA and the need for cesarean section in the univariate analysis (all *p* < 0.05); however, it did not display any significant associations with neonatal or maternal outcomes in the multiple regression models. HbA1c at the end of pregnancy was also associated with macrosomia, LGA and prematurity in the multiple regression analyses (all *p* < 0.05). Finally, maternal treatment requirement was associated with a two-fold higher risk of neonatal hypoglycemia (*p* = 0.032).

No maternal predictor showed a significant association with hospitalization for neonatal complications and jaundice requiring phototherapy in the multiple regression analyses.

### HbA1c stratification

Women participating in this study were further stratified in 2 groups, according to the median HbA1c value at the 1st GDM booking and at the end of pregnancy (< 5.5% vs ≥5.5% (37 mmol/mol). Regarding maternal characteristics, there were significant differences in age, anthropometric and metabolic parameters, high risk ethnicity, cesarean section and medical treatment requirement, when stratifying by HbA1c at 1st GDM booking (See Additional file 3: Table S3). On the other side, few differences in maternal characteristics were found when stratifying by the same HbA1c at the end of pregnancy. Regarding neonatal outcomes, no significant differences were observed when stratifying by HbA1c at the 1st GDM booking, while for HbA1c at the end of pregnancy macrosomia, prematurity and jaundice requiring phototherapy were significantly more prevalent (all *p* < 0.05).

The results of the multiple regression analyses are shown in the Table 4. In women with HbA1c ≥ 5.5% (37 mmol/mol) at 1st GDM booking and at the end of pregnancy, GWG was associated with more macrosomia and LGA (all *p* ≤ 0.03). In women with HbA1c ≥ 5.5% (37 mmol/mol) at the 1st GDM booking, higher prepregnancy BMI was associated with the need for a cesarean section and higher 1-h oGTT glucose values with prematurity (both p ≤ 0.03). In women with an HbA1c ≥ 5.5% (37 mmol/mol) at the end of pregnancy, maternal medical treatment requirement was associated with more hypoglycemia (*p* = 0.020) and less SGA (*p =* 0.044) and prepregnancy BMI was also associated with less SGA (*p* = 0.028). On the other hand, no association was found between maternal predictors and neonatal and maternal complications in the group of women with HbA1c <  5.5% (37 mmol/mol), neither at the 1st GDM booking nor at the end of the pregnancy.

**Table 4:** Maternal predictors of neonatal and maternal outcomes in multiple logistic regression analysis stratified by median HbA1c values

		Odds Ratio	95% CI		*P*-value	Odds Ratio	95% CI		*P*-value
		HbA1c at the 1st GDM booking ≥ 5.5% (37 mmol/mol)				HbA1c at the 1st GDM booking < 5.5% (37 mmol/mol)			
Cesarean section	Prepregnancy BMI (kg/m^2^)	1.07	1.01	1.15	0.031	1.02	0.96	1.09	0.545
Macrosomia^a^	Gestational weight gain (kg)	1.19	1.06	1.33	0.003	0.99	0.85	1.14	0.846
LGA^b^	Gestational weight gain (kg)	1.17	1.07	1.30	0.001	0.99	0.88	1.11	0.813
Prematurity^d^	1-h oGTT glucose value (mmol/l)	1.37	1.03	1.81	0.030	1.10	0.77	1.58	0.590
		HbA1c at the end of pregnancy ≥ 5.5% (37 mmol/mol)^e^				HbA1c at the end of pregnancy < 5.5% (37 mmol/mol)^e^			
Macrosomia^a^	Gestational weight gain (kg)	1.11	1.02	1.21	0.013	-^f^	-^f^	-^f^	-^f^
LGA^b^	Gestational weight gain (kg)	1.09	1.02	1.17	0.009	1.25	0.47	3.29	0.658
SGA^c^	Prepregnancy BMI (kg/m^2^)	0.92	0.86	0.99	0.028	1.06	0.88	1.26	0.547
	Maternal medical treatment requirement	0.50	0.26	0.98	0.044	3.26	0.43	24.75	0.254
Hypoglycemia^g^	Maternal medical treatment requirement	2.52	1.16	5.48	0.020	0.95	0.23	3.91	0.944

Multiple logistic regression analyses with all the variables presented in Table 2, adjusted for maternal age, neonatal sex and gestational age. Median HbA1c values are used for stratification. Predictors are only shown if the *P* value is < 0.05 in the multiple regression analysis in either stratified group. Outcomes are only shown if at least one predictor is found

*Abbreviations*: *HbA1c* Glycated hemoglobin, *CI* Confidence interval, *GDM* Gestational diabetes mellitus, *BMI* Body mass index, *oGTT* Oral glucose tolerance test, *LGA* Large for gestational age, *SGA* Small for gestational age

^a^ birth weight ≥ 4000 g

^b^ LGA: birth weight > 90th percentile for sex and gestational age using the Intergrowth 21st newborn size application tool [30]

^c^ SGA: birth weight < 10th percentile for sex and gestational age using the Intergrowth 21st newborn size application tool [30]

^d^ gestational age < 37 weeks

^e^ this corresponds to the last visit at the GDM clinic

^f^ statistical analysis not possible due to the small amount of outcomes

^g^ capillary or venous glucose value ≤2.5 mmol/l

### Risk stratification at the 1^st^ GDM booking

Finally, probability analyses according to logistic regression models were performed to provide a risk stratification for the 2 most frequent adverse outcomes, i.e. cesarean section and LGA according to the prepregnancy BMI (< 25 vs ≥ 25 kg/m2) and HbA1c (< 5.5 vs ≥ 5.5% (37 mmol/mol)). These predictors can be easily assessed at the 1st GDM booking (Table 5) and were significant in the univariate analyses. In the lowest risk category (BMI <  25 kg/m2 and HbA1c <  5.5%), the probability for cesarean section was 27% (similar to the rate of cesarean section in our tertiary hospital (CHUV) which is 29% [39]) and LGA was 12%, while this risk was 1.8 times higher (48%) for cesarean section and 1.9 times higher (23%) for LGA in the highest risk category.

**Table 5:** Probabilities of future cesarean section and LGA depending on prepregnancy BMI and HbA1c at the 1st GDM booking, according to logistic regression models

Prepregnancy BMI (kg/m^2^)	HbA1c at the 1st GDM booking (%/ mmol/mol))	Cesarean section	LGA^a^
< 25	< 5.5/37	27%	12%
< 25	≥ 5.5/37	36%	14%
≥ 25	< 5.5/37	38%	20%
≥ 25	≥ 5.5/37	48%	23%

*Abbreviations*: *LGA* Large for gestational age, *BMI* Body mass index, *HbA1c* Glycated hemoglobin, *GDM* Gestational diabetes mellitus

^a^ birth weight > 90th percentile for sex and gestational age using the Intergrowth 21st newborn size application tool [30]

## Discussion

In this study of 576 singleton pregnancies in multi-ethnic women with GDM, the following potentially modifiable anthropometric and metabolic parameters were shown to be important predictors of adverse neonatal and/or maternal outcomes (cesarean section, macrosomia, LGA, SGA, hypoglycemia, prematurity) in the multiple regression analyses: prepregnancy BMI, GWG, HbA1c at the end of pregnancy, 1-h oGTT glucose values at GDM diagnosis, and maternal medical treatment requirement. In the presence of GWG, EWG was not associated with adverse neonatal outcomes which hints to the superiority of GWG to predict these outcomes. In terms of future potential clinical risk stratification, the present study also revealed that in the context of a treated population of women with GDM, associations between anthropometric and metabolic parameters and complications are exclusively observed in women with HbA1c ≥ 5.5% (37 mmol/mol). Another risk stratification model showed that in women with a prepregnancy BMI of ≥25 kg/m^2^ and a HbA1c ≥ 5.5% (37 mmol/mol) at the 1st GDM booking, the risk for the two most frequent adverse outcomes, i.e. cesarean section and LGA were around nearly twice as high compared to women with a BMI of < 25 kg/m^2^ and a HbA1c <  5.5% (37 mmol/mol).

Prepregnancy BMI was significantly correlated with the need for cesarean section, which is also consistent with previous studies [40, 41]. It showed an inverse association with SGA in univariate and multiple regression analyses, in good agreement with a metanalysis by Goto et al. and a study by Li et al. [42, 43]. Prepregnancy BMI was associated with LGA in univariate analyses, but these associations did not remain significant in the multiple regression model. This indicates that other correlates, specifically GWG and metabolic control in the 3rd trimester, reflected by the HbA1c at the end of pregnancy, may have a more potent influence on fetal growth. A previous study demonstrated a higher risk for macrosomia and LGA in overweight and obese patients; however these studies did not use a multiple regression model including also the 3rd trimester metabolic control [44]. In accordance with our data, a recent work showed that maternal overweight and obesity are not independent determinants of increased birth weight, in contrast to 3rd trimester glycemic control [21]. Further studies are necessary to elucidate this observation.

GWG was a significant predictor of LGA and macrosomia in both univariate and multiple regression analyses. This is in accordance with previous studies, which demonstrate a direct association between maternal weight gain during pregnancy and offspring birthweight [12, 45–48]. Importantly, in our population most of the weight was gained before the 1st visit at the GDM clinic which occurred at a mean of 29 weeks of GA (10.3 ± 5.6 vs 2.5 ± 2.7 kg after). This points out the importance for GWG monitoring early in pregnancy, as rapid weight gain in early pregnancy may be associated with the risk of offspring complications. Although EWG showed a significant association with LGA and macrosomia in univariate analyses, these associations did not remain significant in the multiple regression model, suggesting that absolute GWG may influence fetal weight gain more decidedly. EGW was shown to be a significant predictor of LGA and macrosomia in former studies [14, 44, 49, 50], but these studies did not include GWG in their model.

In the multiple regression model, the 1-h oGTT glucose value predicted the need for cesarean section. There were no other significant associations between oGTT values and neonatal or maternal complications in this treated population. Similarly, a recent study by Ott et al., also found that glucose value at oGTT was not correlated with birth outcomes in treated populations, with few exceptions [21]. These findings are in contrast with the large-scale Hyperglycemia and adverse pregnancy outcome study (HAPO) study [26] that showed a continuous association between maternal glucose values at the oGTT and fetal overgrowth in an untreated population. The discrepancy in our results may be due to the treatment’s intensity and efficacy in the setting of a clinical follow-up.

Moreover, newborns whose mothers required medical treatment presented hypoglycemia twice as often, despite the rigorous feeding protocol. In contrast, in a recent Atlantic DIP study there was no difference in hypoglycemia rates in newborns of patients with GDM treated with insulin vs medical nutritional care [28]. The higher occurrence of neonatal hypoglycemia in the context of a maternal treatment may imply a worse glycemic control, but also higher variability and glucose peaks in these patients, which is not always captured by capillary glycemia and the HbA1c. To the best of our knowledge, this is a novel observation. A possible bias in this observation may be linked to the more frequent glucose monitoring in the newborns whose mothers required a medical treatment.

In the multiple regression analyses, HbA1c at the 1st GDM booking did not reveal any association with neonatal and maternal outcomes, in good agreement with previous studies [17, 18, 51, 52]. This finding could be attributed to the fact that HbA1c reflects the metabolic control of the preceding weeks to months which is less relevant at around 29 weeks of gestational age. It is also explicable to the subsequent treatment’s efficacy and metabolic control. Nevertheless, HbA1c at 1st GDM booking was associated with several outcomes in univariate analyses (cesarean section, macrosomia, LGA). Furthermore, when HbA1c at the 1st GDM visit was < 5.5%, there were no significant correlations between predictors and adverse outcomes. We also showed that HbA1c is useful for the stratification of the risk of LGA and cesarean section at the 1st GDM booking.

By contrast, HbA1c at the end of pregnancy was a powerful predictor of preterm birth, macrosomia and LGA in the multiple regression analyses. The HbA1c at the end of pregnancy value reflects the glycemic control during the weeks before birth, which is a period of rapid fetal weight gain. Few studies have evaluated the correlation between 3rd trimester HbA1c and anthropometric parameters as well as neonatal outcomes. Barquiel et al. showed that 3rd trimester HbA1c > 5% (31 mmol/mol) increases the risk of LGA and other neonatal complications [22]. Mikkelsen et al. found that in women with GDM, HbA1c > 5.6% (38 mmol/mol) is associated with a threefold increase of LGA and six-fold increase of neonatal hypoglycemia. Gandhi et al. demonstrated a higher mean birth weight in newborns of women with GDM with a 3rd trimester HbA1c > 6.5% (48 mmol/mol) [24]. To the best of our knowledge, this is the first study that discovered an association between HbA1c at the end of pregnancy and prematurity. Tight 3rd trimester glycemia control seems to be a key target for avoiding neonatal complications in pregnancies with GDM.

We conducted a risk stratification model based on the median HbA1c values at 1st GDM booking and at the end of pregnancy (cut-off of 5.5% (37 mmol/mol) for both). Regarding maternal characteristics, there were significant differences in anthropometric and metabolic parameters, high risk ethnicity, medical treatment requirement and cesarean section, when stratifying by HbA1c at 1st booking (< 5.5% vs ≥5.5% (37 mmol/mol)). Few differences were found when stratifying by HbA1c at the end of pregnancy. Regarding neonatal outcomes no significant differences were observed when stratifying by HbA1c at the 1st booking, while for HbA1c at the end of pregnancy macrosomia, prematurity and jaundice requiring phototherapy were more prevalent when HbA1c was ≥5.5% (37 mmol/mol) (all *p < 0.05*). Of note, achieving an HbA1c of < 5.5% (37 mmol/mol) needed many lifestyle advices and encouragement and in almost half of the women (46%) also a medical treatment. We only have national Swiss data for cesarean section rates and prematurity: Comparing to these data, cesarean section rates in the subgroup with HbA1c < 5.5% (37 mmol/mol) at the 1st booking, were comparable to the general population (31% vs 29%), but were higher for the other subgroups [39]. Prematurity was lower in the subgroup with HbA1c < 5.5% (37 mmol/mol) at the end of the pregnancy compared to the general population (1.2% vs 7%) [53]. When comparing to international and mainly US data, macrosomia and jaundice requiring phototherapy were even similar in both HbA1c-subgroups [54, 55]. In the subgroup of HbA1c < 5.5% (37 mmol/mol) at the end of pregnancy, hospitalization for neonatal complications was similar to international data [56, 57]. While it is encouraging to observe that many outcomes in this treated GDM population are close to the general population, it is important to note that ethnic and population differences as well as differences in the clinical settings also play a role.

Interestingly, the associations between maternal predictors and neonatal and maternal outcomes were exclusively seen when HbA1c was ≥5.5% (37 mmol/mol). This may highlight the need for a more rigorous follow-up in these women, and a tighter monitoring of their glucose values. In these women, a repeated HbA1c 1–2 months later could be discussed if capillary glucose values were normal. In cases where the HbA1c value remains high despite normal capillary values, continuous glucose monitoring could be proposed if occasional peaks or nocturnal glucose elevation are suspected. Further studies are necessary to evaluate whether HbA1c could be used for risk stratification of adverse neonatal and maternal outcomes in pregnancies complicated with GDM, and to determine an HbA1c threshold above which maternal or neonatal follow-up may need to be intensified.

Finally, we performed probability analyses according to logistic regression models for risk stratification at the 1st booking based on simple anthropometric and metabolic parameters, such as maternal prepregnancy BMI and HbA1c at the 1st booking using the two most frequent adverse outcomes (caesarean section and LGA). In the highest risk category (BMI of ≥25 kg/m^2^ and a HbA1c ≥ 5.5% (37 mmol/mol)), the probability was 1.8 times higher and 1.9 times higher for cesarean section and LGA respectively compared to the lowest category. We are not aware of other studies providing a risk stratification for these clinically relevant parameters.

The strengths of our study included its prospective nature, which ensured the presence of complete detailed information on maternal and neonatal characteristics, as well as several novel findings in the multiple regression analyses, the stratified analyses and the probability analyses. However, several limitations may also be noted. Firstly, HbA1c is subject to specific pregnancy changes, shows ethnic variation, and may be affected by conditions such as haemolytic anaemia, chronic renal failure, severe liver disease and anaemia of chronic disease, which might influence its validity [58]. HbA1c at the end of pregnancy was only documented after March 2015, leading to a smaller sample group for this predictor (*n* = 201), which nevertheless did not limit its statistical significance. However, a higher sample size could have identified its predictive role for even more outcomes. A larger population would also have led to a higher number of rare outcomes and therefore would have ensured the possibility to include these outcomes in the multivariate models. Moreover, the indication for cesarean section was not specified. Dividing the population in cesarean section subgroups, such as elective or emergency could be interesting, but would lead to smaller population sizes and limited statistical power. Another limitation is that the prepregnancy BMI and the gestational weight gain were determined using the self-reported pre-pregnancy weight or data from the medical charts. A recent review study showed that the magnitude of error of the self-reported weight is small, and does not largely bias associations between pregnancy-related weight and birth outcomes [59]. In our study, prepregnancy BMI and BMI at the 1st booking after GDM diagnosis were highly correlated (*r* = 0.93). Identification of modifiable factors before GDM diagnosis or even before pregnancy would be essential to improve maternal and neonatal outcomes, but unfortunately we only have valid data for women after the diagnosis of GDM. Another limitation is that this is an explorative study. Its results can be used to create new hypotheses.

## Conclusions

The present study demonstrated the important role of simple anthropometric and metabolic maternal predictors on the occurrence of adverse neonatal and maternal outcomes. We were able to identify several very important predictors: prepregnancy BMI, GWG, maternal treatment requirement and HbA1c at the end of pregnancy. We also found that predictors were exclusively associated to adverse neonatal and maternal outcomes when the HbA1c values were ≥ 5.5% (37 mmol/mol) at the 1st GDM booking and/or at the end of the pregnancy. Risk stratification depending on the maternal prepregnancy BMI and HbA1c at the 1st GDM booking may allow for risk-adapted care in patients with GDM.

## Additional files

Additional file 1: **Table S1.** (Supplementary to Table 2) – Maternal predictors of adverse neonatal and maternal outcomes in univariate analysis.

Additional file 2: **Table S2.** (Supplementary to Table 3) - Maternal predictors of adverse neonatal and maternal outcomes in stepwise multiple logistic regression analysis.

Additional file 3: **Table S3.** Comparisons between maternal and neonatal characteristics stratified by median HbA1c values.

## Data Availability

The databases used and analyzed are available from the corresponding author on reasonable request.

## Abbreviations

BMI: Body mass index
CI: Confidence interval
DCCT: Diabetes control and complications trial
EGW: Excessive weight gain
GDM: Gestational diabetes mellitus
GWG: Gestational weight gain
HAPO: Hyperglycemia and adverse pregnancy outcome study
HbA1c: Glycated hemoglobin
HPLC: High-performance liquid chromatography
ICU: Intensive care unit
IFCC: International federation of clinical chemistry and laboratory medicine
IOM: Institute of medicine
LGA: Large for gestational age
oGTT: Oral glucose tolerance test
SGA: Small for gestational age
