# Supplementary material for: Potentially modifiable predictors of adverse neonatal and maternal outcomes in pregnancies with gestational diabetes mellitus: can they help for future risk stratification and risk-adapted patient care?
Source: BMC Pregnancy Childbirth. 2019 Dec 4;19:469. doi: 10.1186/s12884-019-2610-2 (PMC6894261; doi:10.1186/s12884-019-2610-2)
Supplement: Supplementary file 1 — Table S1. (Supplementary to Table 2) – Maternal predictors of adverse neonatal and maternal outcomes in univariate analysis. [file 12884_2019_2610_MOESM1_ESM.docx]

Additional file 1: Table S1 (Supplement to Table 2) – Maternal predictors of adverse neonatal and maternal outcomes in univariate analysis.

| Presence of neonatal and maternal outcomes | Maternal predictors | Odds Ratio | 95% CI | | p-value |
| --- | --- | --- | --- | --- | --- |
| Cesarean section | Prepregnancy BMI (kg/m^2^) | 1.06 | 1.02 | 1.09 | 0.001 |
|  | Gestational weight gain (kg) | 1.01 | 0.98 | 1.03 | 0.573 |
|  | Excess weight gain ^1^ | 1.18 | 1.00 | 1.38 | 0.048 |
|  | Fasting oGTT glucose (mmol/l) | 1.12 | 0.89 | 1.42 | 0.369 |
|  | 1-hour oGTT glucose (mmol/l) | 1.10 | 0.99 | 1.23 | 0.084 |
|  | 2-hour oGTT glucose (mmol/l) | 1.08 | 0.97 | 1.20 | 0.166 |
|  | HbA1c at the 1st GDM booking (%/mmol/mol) | 1.63 | 1.04 | 2.56 | 0.033 |
|  | HbA1c at the end of pregnancy (%/mmol/mol)^3^ | 1.01 | 0.44 | 2.32 | 0.987 |
|  | Maternal medical treatment requirement | 1.65 | 1.11 | 2.44 | 0.013 |
| Macrosomia ^2^ | Prepregnancy BMI (kg/m^2^) | 1.03 | 0.98 | 1.09 | 0.242 |
|  | Gestational weight gain (kg) | 1.11 | 1.05 | 1.17 | <0.001 |
|  | Excess weight gain ^1^ | 1.41 | 1.10 | 1.79 | 0.006 |
|  | Fasting oGTT glucose (mmol/l) | 1.63 | 1.16 | 2.29 | 0.005 |
|  | 1-hour oGTT glucose (mmol/l) | 1.17 | 0.96 | 1.42 | 0.115 |
|  | 2-hour oGTT glucose (mmol/l) | 1.21 | 0.99 | 1.47 | 0.065 |
|  | HbA1c at the 1st GDM booking (%/mmol/mol) | 2.92 | 1.42 | 6.03 | 0.004 |
|  | HbA1c at the end of pregnancy (%/mmol/mol)^3^ | 6.45 | 1.82 | 22.90 | 0.004 |
|  | Maternal medical treatment requirement | 2.75 | 1.29 | 5.86 | 0.009 |
| LGA ^4^ | Prepregnancy BMI (kg/m^2^) | 1.04 | 1.00 | 1.08 | 0.064 |
|  | Gestational weight gain(kg) | 1.08 | 1.04 | 1.12 | <0.001 |
|  | Excess weight gain ^1^ | 1.46 | 1.23 | 1.74 | <0.001 |
|  | Fasting oGTT glucose (mmol/l) | 1.46 | 1.12 | 1.91 | 0.006 |
|  | 1-hour oGTT glucose (mmol/l) | 1.13 | 0.99 | 1.29 | 0.077 |
|  | 2-hour oGTT glucose (mmol/l) | 1.06 | 0.93 | 1.22 | 0.364 |
|  | HbA1c at the 1st GDM booking (%/mmol/mol) | 1.84 | 1.08 | 3.14 | 0.025 |
|  | HbA1c at the end of pregnancy (%/mmol/mol)^3^ | 2.84 | 1.01 | 7.93 | 0.047 |
|  | Maternal medical treatment requirement | 1.91 | 1.14 | 3.20 | 0.014 |
| SGA ^5^ | Prepregnancy BMI (kg/m^2^) | 0.95 | 0.81 | 1.00 | 0.077 |
|  | Gestational weight gain (kg) | 0.99 | 0.95 | 1.03 | 0.663 |
|  | Excess weight gain ^1^ | 0.82 | 0.61 | 1.11 | 0.201 |
|  | Fasting oGTT glucose (mmol/l) | 0.94 | 0.63 | 1.39 | 0.747 |
|  | 1-hour oGTT glucose (mmol/l) | 1.00 | 0.84 | 1.19 | 0.987 |
|  | 2-hour oGTT glucose (mmol/l) | 1.09 | 0.93 | 1.29 | 0.283 |
|  | HbA1c at the 1st GDM booking (%/mmol/mol) | 0.76 | 0.37 | 1.58 | 0.467 |
|  | HbA1c at the end of pregnancy (%/mmol/mol)^3^ | 1.05 | 0.25 | 4.42 | 0.950 |
|  | Maternal medical treatment requirement | 0.59 | 0.32 | 1.08 | 0.087 |
| Hypoglycemia ^6^ | Prepregnancy BMI (kg/m^2^) | 1.00 | 0.95 | 1.06 | 0.851 |
|  | Gestational weight gain (kg) | 1.00 | 0.96 | 1.04 | 0.916 |
|  | Excess weight gain ^1^ | 1.20 | 0.96 | 1.50 | 0.107 |
|  | Fasting oGTT glucose (mmol/l) | 0.97 | 0.65 | 1.43 | 0.866 |
|  | 1-hour oGTT glucose (mmol/l) | 1.04 | 0.88 | 1.23 | 0.648 |
|  | 2-hour oGTT glucose (mmol/l) | 1.11 | 0.94 | 1.32 | 0.216 |
|  | HbA1c at the 1st GDM booking (%/mmol/mol) | 1.33 | 0.68 | 2.61 | 0.398 |
|  | HbA1c at the end of pregnancy (%/mmol/mol)^3^ | 0.55 | 0.15 | 1.96 | 0.354 |
|  | Maternal medical treatment requirement | 2.03 | 1.06 | 3.88 | 0.032 |
| Prematurity ^7^ | Prepregnancy BMI (kg/m^2^) | 0.96 | 0.90 | 1.03 | 0.228 |
|  | Gestational weight gain (kg) | 0.97 | 0.94 | 1.01 | 0.159 |
|  | Excess weight gain ^1^ | 0.92 | 0.68 | 1.24 | 0.570 |
|  | Fasting oGTT glucose (mmol/l) | 1.01 | 0.66 | 1.54 | 0.961 |
|  | 1-hour oGTT glucose (mmol/l) | 1.13 | 0.94 | 1.36 | 0.188 |
|  | 2-hour oGTT glucose (mmol/l) | 1.05 | 0.88 | 1.25 | 0.569 |
|  | HbA1c at the 1st GDM booking (%/mmol/mol) | 1.15 | 0.49 | 2.67 | 0.748 |
|  | HbA1c at the end of pregnancy (%/mmol/mol)^3^ | 12.48 | 1.85 | 84.13 | 0.010 |
|  | Maternal medical treatment requirement | 1.21 | 0.56 | 2.62 | 0.628 |
| Hospitalization for neonatal complication | Prepregnancy BMI (kg/m^2^) | 1.01 | 0.96 | 1.07 | 0.756 |
|  | Gestational weight gain (kg) | 0.97 | 0.93 | 1.00 | 0.082 |
|  | Excess weight gain ^1^ | 1.05 | 0.82 | 1.36 | 0.695 |
|  | Fasting oGTT glucose (mmol/l) | 0.84 | 0.55 | 1.28 | 0.419 |
|  | 1-hour oGTT glucose (mmol/l) | 1.09 | 0.93 | 1.28 | 0.306 |
|  | 2-hour oGTT glucose (mmol/l) | 1.06 | 0.90 | 1.24 | 0.489 |
|  | HbA1c at the 1st GDM booking (%/mmol/mol) | 1.50 | 0.74 | 3.02 | 0.261 |
|  | HbA1c at the end of pregnancy (%/mmol/mol)^3^ | 0.77 | 0.17 | 3.59 | 0.741 |
|  | Maternal medical treatment requirement | 0.91 | 0.47 | 1.78 | 0.785 |
| Jaundice requiring phototherapy | Prepregnancy BMI (kg/m^2^) | 1.08 | 0.99 | 1.18 | 0.071 |
|  | Gestational weight gain (kg) | 1.06 | 0.96 | 1.16 | 0.249 |
|  | Excess weight gain ^1^ | 1.31 | 0.89 | 1.95 | 0.174 |
|  | Fasting oGTT glucose (mmol/l) | 1.08 | 0.59 | 2.00 | 0.817 |
|  | 1-hour oGTT glucose (mmol/l) | 1.08 | 0.82 | 1.41 | 0.581 |
|  | 2-hour oGTT glucose (mmol/l) | 1.03 | 0.79 | 1.35 | 0.828 |
|  | HbA1c at the 1st GDM booking (%/mmol/mol) | 1.45 | 0.44 | 4.77 | 0.541 |
|  | HbA1c at the end of pregnancy (%/mmol/mol)^3^ | 0.02 | 0.00001 | 41.01 | 0.328 |
|  | Maternal medical treatment requirement | 0.84 | 0.26 | 2.74 | 0.772 |
| 5-min Apgar score <7 | Prepregnancy BMI (kg/m^2^) | 0.93 | 0.79 | 1.08 | 0.349 |
|  | Gestational weight gain (kg) | 0.98 | 0.91 | 1.06 | 0.670 |
|  | Excess weight gain ^1^ | 0.71 | 0.31 | 1.62 | 0.416 |
|  | Fasting oGTT glucose (mmol/l) | 0.98 | 0.41 | 2.34 | 0.966 |
|  | 1-hour oGTT glucose (mmol/l) | 0.99 | 0.68 | 1.44 | 0.964 |
|  | 2-hour oGTT glucose (mmol/l) | 1.12 | 0.77 | 1.61 | 0.553 |
|  | HbA1c at the 1st GDM booking (%/mmol/mol) | 0.68 | 0.09 | 5.28 | 0.709 |
|  | HbA1c at the end of pregnancy (%/mmol/mol)^3^ | 0.27 | 0.007 | 9.89 | 0.476 |
|  | Maternal medical treatment requirement | 0.78 | 0.15 | 4.15 | 0.771 |

Abbreviations: *CI* confidence interval, *BMI* body mass index, *GDM* gestational diabetes mellitus, o*GTT* oral glucose tolerance test, *HbA1c* glycated hemoglobin, *LGA* Large for gestational age, *SGA* Small for gestational age,

^1^ according to the Institute of Medicine 2009 guidelines [13]

^2^ birth weight ≥4000gr

^3^ this corresponds to the last visit at the GDM clinic

^4^ LGA: birth weight >90th percentile for sex and gestational age using the Intergrowth 21^st^ newborn size application tool [30]

^5^ SGA: birth weight <10th percentile for sex and gestational age using the Intergrowth 21^st^ newborn size application tool [30]

^6^ capillary or venous glucose value ≤ 2.5 mmol/l.

^7^ gestational age <37 weeks.

Univariate logistic regression analyses adjusted for maternal age, neonatal sex and gestational age. This table shows the odds ratio for the presence (vs absence) of adverse neonatal and maternal outcomes.
