# Supplementary material for: Potentially modifiable predictors of adverse neonatal and maternal outcomes in pregnancies with gestational diabetes mellitus: can they help for future risk stratification and risk-adapted patient care?
Source: BMC Pregnancy Childbirth. 2019 Dec 4;19:469. doi: 10.1186/s12884-019-2610-2 (PMC6894261; doi:10.1186/s12884-019-2610-2)
Supplement: Supplementary file 2 — Table S2. (Supplementary to Table 3) - Maternal predictors of adverse neonatal and maternal outcomes in stepwise multiple logistic regression analysis. [file 12884_2019_2610_MOESM2_ESM.docx]

Additional file 2: Table S2 (supplementary to table 3) - Maternal predictors of adverse neonatal and maternal outcomes in stepwise multiple logistic regression analysis.

|  |  | Odds Ratio | 95% CI | | p-value |
| --- | --- | --- | --- | --- | --- |
| Cesarean section | Prepregnancy BMI (kg/m^2^) | 1.05 | 1.01 | 1.09 | 0.022 |
|  | Excess weight gain ^1^ |  |  |  | 0.416 |
|  | 1-hour oGTT glucose (mmol/l) | 1.15 | 1.02 | 1.29 | 0.024 |
|  | 2-hour oGTT glucose (mmol/l) |  |  |  | 0.920 |
|  | HbA1c at the 1^st^ GDM booking (%/mmol/mol) |  |  |  | 0.140 |
|  | Maternal medical treatment requirement |  |  |  | 0.428 |
| Macrosomia ^2^ | Gestational weight gain (kg) | 1.11 | 1.05 | 1.19 | 0.001 |
|  | Excess weight gain^1^ |  |  |  | 0.784 |
|  | Fasting oGTT glucose (mmol/l) |  |  |  | 0.657 |
|  | 1-hour oGTT glucose (mmol/l) |  |  |  | 0.764 |
|  | 2-hour oGTT glucose (mmol/l) |  |  |  | 0.119 |
|  | HbA1c at the 1^st^ GDM booking (%/mmol/mol) |  |  |  | 0.288 |
|  | HbA1c at the end of pregnancy (%/mmol/mol) ^3^ | 6.84 | 1.53 | 30.54 | 0.012 |
|  | Maternal medical treatment requirement |  |  |  | 0.381 |
| LGA ^4^ | Prepregnancy BMI (kg/m^2^) |  |  |  | 0.053 |
|  | Gestational weight gain(kg) | 1.11 | 1.06 | 1.17 | <0.001 |
|  | Excess weight gain ^1^ |  |  |  | 0.515 |
|  | Fasting oGTT glucose (mmol/l) |  |  |  | 0.872 |
|  | 1-hour oGTT glucose (mmol/l) |  |  |  | 0.515 |
|  | HbA1c at the 1^st^ GDM booking (%/mmol/mol) |  |  |  | 0.224 |
|  | HbA1c at the end of pregnancy (%/mmol/mol)^3^ | 4.68 | 1.27 | 17.28 | 0.021 |
|  | Maternal medical treatment requirement |  |  |  | 0.663 |
| SGA ^5^ | Prepregnancy BMI (kg/m^2^) | 0.93 | 0.88 | 0.99 | 0.038 |
|  | Maternal medical treatment requirement |  |  |  | 0.145 |
| Hypoglycemia ^6^ | Excess weight gain ^1^ |  |  |  | 0.446 |
|  | Maternal medical treatment requirement | 2.03 | 1.06 | 3.88 | 0.032 |
| Prematurity ^7^ | Gestational weight gain (kg) |  |  |  | 0.114 |
|  | 1-hour oGTT glucose (mmol/l) |  |  |  | 0.380 |
|  | HbA1c at the end of pregnancy(%/mmol/mol) ^3^ | 22.4 | 2.36 | 213.2 | 0.007 |
| Hospitalization for neonatal complication | Gestational weight gain (kg) |  |  |  | 0.082 |
| Jaundice requiring phototherapy | BMI at the 1^st^ GDM booking (kg/m^2^) |  |  |  | 0.063 |
|  | Excess weight gain ^1^ |  |  |  | 0.607 |

Abbreviations: *CI* confidence interval, *BMI* body mass index, *GDM* gestational diabetes mellitus, o*GTT* oral glucose tolerance test, *HbA1c* glycated hemoglobin, *LGA* Large for gestational age, *SGA* Small for gestational age

^1^ according to the Institute of Medicine guidelines [13]

^2^ birth weight ≥4000gr

^3^ this corresponds to the last visit at the GDM clinic

^4^ LGA: birth weight >90th percentile for sex and gestational age using the Intergrowth 21^st^ newborn size application tool [[6](#_ENREF_2)])

^5^ SGA: birth weight <10th percentile for sex and gestational age using the Intergrowth 21^st^ newborn size application tool [[6](#_ENREF_2)]

^6^ capillary or venous glucose value ≤ 2.5 mmol/l.

^7^ gestational age <37 weeks.

Stepwise multiple logistic regression analyses with all the variables presented in table 2, adjusted for maternal age, neonatal sex and gestational age.
