# Supplementary material for: Potentially modifiable predictors of adverse neonatal and maternal outcomes in pregnancies with gestational diabetes mellitus: can they help for future risk stratification and risk-adapted patient care?
Source: BMC Pregnancy Childbirth. 2019 Dec 4;19:469. doi: 10.1186/s12884-019-2610-2 (PMC6894261; doi:10.1186/s12884-019-2610-2)
Supplement: Supplementary file 3 — Table S3. Comparisons between maternal and neonatal characteristics stratified by median HbA1c values. [file 12884_2019_2610_MOESM3_ESM.docx]

Additional file 3: Table S3-Comparisons between maternal and neonatal characteristics stratified by median HbA1c values

| **Maternal characteristics** | HbA1c at the 1st booking <5.5%  (37mmol/mol) | HbA1c at the 1st booking ≥5.5%  (37mmol/mol) | p-value | HbA1c at the end of pregnancy <5.5%*  (37mmol/mol) | HbA1c at the end of pregnancy ≥5.5%*  (37mmol/mol) | p-value |
| --- | --- | --- | --- | --- | --- | --- |
| Age (years) | 32.4±5.2 | 33.1±5.7 | 0.041 | 33.1±4.7 | 32.8±5.6 | 0.982 |
| Prepregnancy BMI (kg/m^2^) | 25.1±5.4 | 26.9.±5.4 | <0.001 | 26.1.±4.7 | 26.1.±5.5 | 0.421 |
| BMI at the 1^st^ GDM booking (kg/m^2^) | 28.8±5.2 | 31.1±5.5 | <0.001 | 30.0±4.6 | 30.0±5.6 | 0.257 |
| Gestational weight gain (kg) | 11.7±7.9 | 13.4±6.4 | 0.007 | 12.3±5.3 | 12.7±7.5 | 0.241 |
| Gestational weight gain until the 1^st^ GDM booking (kg) | 9.5±5.5 | 11.0±5.7 | 0.002 | 10.2±4.9 | 10.3±5.8 | 0.495 |
| Excessive gestational weight gain^1^ n(%) | 62(24) | 110(35) | 0.003 | 17(21) | 155(32) | 0.122 |
| Fasting oGTT glucose value (mmol/l) | 5.0±0.6 | 5.2±0.8 | <0.001 | 5.0±0.6 | 5.2±0.8 | 0.011 |
| 1-hour oGTT glucose value (mmol/l) | 9.3±1.9 | 9.9±1.9 | <0.001 | 9.5±2.0 | 9.7±1.9 | 0.308 |
| 2-hour oGTT glucose value (mmol/l) | 7.6±1.8 | 8.0±2.0 | 0.017 | 7.5±1.8 | 7.8±1.9 | 0.022 |
| High risk ethnicity n(%) | 86(34) | 133(44) | 0.006 | 16(20) | 203(43) | 0.006 |
| Cesarean section n(%) | 79(31) | 133(44) | 0.005 | 30(38) | 132(38) | 0.743 |
| Maternal medical treatment requirement n(%) | 108 (46) | 189 (68) | <0.001 | 37 (46) | 260 (60) | 0.017 |
|  |  |  |  |  |  |  |
| **Neonatal characteristics** |  |  |  |  |  |  |
| LGA^2^ n(%) | 39(15) | 56(18) | 0.171 | 9(10.8) | 86(17.5) | 0.258 |
| SGA^3^ n(%) | 28(11) | 26(8.3) | 0.302 | 7(8.4) | 47(9.4) | 0.486 |
| Macrosomia^4^ n(%) | 17(6.5) | 28(9.0) | 0.160 | 3(3.6) | 42(8.5) | 0.011 |
| Hypoglycemia^5^n(%) | 23(9.8) | 33(11) | 0.393 | 11(14.9) | 45(10.0) | 0.440 |
| Prematurity^6^ n(%) | 19(7.2) | 28(9.0) | 0.479 | 1(1.2) | 46(9.4) | 0.001 |
| Hospitalization for neonatal complication n(%) | 20(8.5) | 41(14) | 0.236 | 5(6.8) | 56(12.4) | 0.138 |
| Jaundice requiring phototherapy n(%) | 7(2.7) | 14(4.5) | 0.625 | 1(1.2) | 20(4.1) | 0.045 |
| Apgar 5-min<7 n(%) | 4(1.5) | 6(1.9) | 0.719 | 2(2.4) | 8(1.6) | 0.639 |

Abbreviations: *BMI* body mass index, *GDM* gestational diabetes mellitus, o*GTT* oral glucose tolerance test, *HbA1c* glycated hemoglobin, *LGA* Large for gestational age, *SGA* Small for gestational age.

*less data are available for HbA1c at the end of pregnancy

^1^ according to the Institute of Medicine guidelines [13]

^2^ LGA: birth weight >90th percentile for sex and gestational age using the Intergrowth 21^st^ newborn size application tool [30]

^3^ SGA: birth weight <10th percentile for sex and gestational age using the Intergrowth 21^st^ newborn size application tool [30]

^4^ birth weight ≥4000gr

^5^ capillary or venous glucose value ≤ 2.5 mmol/l.

^6^ gestational age <37 weeks.

Comparisons made using the unpaired t-test for continuous variables and the Fischer’s exact test for binary variables.
